# A stakeholder analysis of the perceived outcomes of developing and implementing England’s obesity strategy 2008–2011

**DOI:** 10.1186/1471-2458-14-441

**Published:** 2014-05-10

**Authors:** Corinna Hawkes, Amy L Ahern, Susan A Jebb

**Affiliations:** 1 MRC Human Nutrition Research, Elsie Widdowson Laboratory, Fulbourn Road, Cambridge, UK; 2 Current affiliation WCRF International, 22 Bedford Square, London, UK; 3 Current address: Nuffield Department of Primary Care Health Sciences, University of Oxford, Radcliffe Observatory Quarter, Woodstock Road, Oxford OX2 6GG, UK

**Keywords:** Obesity, Strategy, Policy, Population-based, Qualitative, Stakeholder perceptions, England, Evaluation, Monitoring

## Abstract

**Background:**

International recommendations urge governments to implement population-based strategies to reduce the burden of obesity. This study assesses the development and implementation of the obesity strategy in England 2008–2011, *Healthy Weight, Healthy Lives (HWHL).* The aim was to identify if stakeholders perceived *HWHL* to have made any difference to the action to address obesity in England, with the ultimate objective of identifying insights that could inform the development and implementation of future obesity strategies in England and elsewhere.

**Methods:**

Qualitative study using semi-structured interviews and thematic framework analysis. 40 stakeholders involved in the development and implementation of the obesity strategy were interviewed.

**Results:**

Evidence from this study suggests that *HWHL* was perceived to have made a positive difference to efforts to address obesity in England. It was credited with creating political buy-in, engaging more stakeholders, stimulating and facilitating action, enhancing knowledge and changing attitudes. But it was reported to have failed to fully catalyse action across all government departments and sectors, or to develop adequate mechanisms for learning about the effectiveness of the different elements and actions in the Strategy. Key elements of the Strategy contributing towards to the perceived positive differences included its multi-faceted, inclusive nature; governance structures; monitoring programme to assess progress against national and local targets; child-focus; and funding. The development of the Strategy was said to be stimulated and aided by the prior synthesis of a critical mass of scientific evidence.

**Conclusions:**

The English experience of *HWHL* lends support to the recommendations to develop population-based obesity strategies. It indicates that in order to stimulate comprehensive, inter-sectoral action, obesity strategies need to take a population-based, multi-faceted approach, be implemented through a clear governance structure, follow a systematic process of aligning goals, objectives and agendas between government departments and sectors with a stake in obesity, and have a clear system of reporting changes in obesity rates against a target. In order to design effective policies and to build the case for continued investment, obesity strategies also need to incorporate a national framework for learning and evaluation from the outset.

## Background

### Introduction and objective

Around the world, the public health burden of overweight and obesity is placing pressure on health and national resources. To tackle obesity, leading health experts, including those convened by the World Health Organization (WHO) [1], recommend that governments develop and implement “population-based strategies” [2].

The call for population-based strategies reflects the evidence that the causes of obesity are complex and multifaceted and require a range of different solutions at multiple levels and in multiple sectors [2-6]. Given the high prevalence of excess weight gain, it is also unlikely to be practical or cost effective to rely solely on individual-level interventions, though these are a necessary component for the treatment of individuals with established obesity.

In 2010 the World Health Organization (WHO) estimated that 38% of countries around the world had implemented and funded obesity strategies, policies or plans [7]. Yet there has been little published research into the development or impact of these strategies [8]. In England, the first national obesity strategy *Healthy Weight, Healthy Lives: A Cross Government Strategy for England* (2008–2011) was published in 2008 [9]. Beyond some specific evaluations of individual programmes [10], there has been little published about the development of the Strategy. There has been no analysis of the impact of the Strategy as a whole, or of what lessons can be learned from the experience.

This study aims to identify the perceived outcomes of *Healthy Weight, Healthy Lives* (*HWHL*) on public health efforts to address obesity in England and the key elements responsible for these outcomes. It assesses whether *HWHL* was perceived to have made any difference on efforts to address obesity in England, with the ultimate objective of identifying insights that could inform the development and implementation of future obesity strategies in England and elsewhere. The study is based on the views of stakeholders involved in the development and implementation of the Strategy. Given the complexity of obesity, the multiple actors and diverse components within the Strategy, this research does not aim to ascertain to what extent any changes in obesity rates could be attributed specifically to *HWHL*.

### About *Healthy Weight, Healthy Lives*

A continuum of official reports were published on obesity in England through the 2000s. These reports described the increasing prevalence of obesity and were critical of the government response [11-13]. In 2007, the “Foresight” report on obesity, prepared by the government’s Chief Scientific Adviser [6] made clear the urgent need to take comprehensive, co-ordinated action. That same year, the government revised the national obesity target [14], requested Primary Care Trusts (the National Health Service bodies responsible for population health improvement locally) to develop obesity plans [15], supported the development of a National Obesity Observatory (NOO) [16], and established the National Child Measurement Programme (NCMP) [17].

It was in this context that the government published *Healthy Weight, Healthy Lives* (*HWHL)* as its overarching strategy to deal with the problem. Taking the population-based approach recommended in the Foresight report, it addressed the different causes of obesity. Drafted with five different “streams”, it brought together actions already underway and policies already implemented, as well as new initiatives and programmes. It focused largely on prevention, especially (although not only) among children, but also included obesity management.

*HWHL* was an explicitly cross-government strategy, published jointly by the Department of Health and the Department of Children, Schools and Families (now the Department for Education) following consultation with other government departments and stakeholders. It set out actions for actors outside the health sector and the roles and responsibilities of other government departments (Table 1). *HWHL* adopted the government target set the previous year: to reduce the proportion of overweight and obese children to 2000 levels by 2020.

**Table 1:** **The five streams of *Healthy Weight, Healthy Lives***

**Stream**	**Core actions and programmes implemented**	**Main cross-sectoral stakeholder**
Children: healthy growth and healthy weight	Breastfeeding initiatives “Healthy Schools” programme*	Education departments and/or children’s departments
Promoting healthier food choices	Healthy Food Code	Food Standards Agency
Building physical activity into our lives	Active Travel “Healthy Towns” programme**	Transport, planning and/or environment departments
Personalized advice and support	Change4Life, NHS Choices website, Weight management services at the local level	Private sector, Weight management professionals
Creating incentives for better health***	Reported not to be implemented to a significant degree	

*A government programme designed to promote a “whole school approach” to health. Schools could receive accreditation for being a “Healthy School”. It was discontinued in 2012.

**A pilot scheme to provide specific towns/boroughs with resources to take holistic, environmental approaches through infrastructure improvements to address obesity.

***Aimed to ensure there were stronger incentives in place for people, companies and the National Health Service to invest in health.

*HWHL* was implemented at all levels of government with £372 million in funding. At a national level, it was managed by a formal Cross Government Obesity Unit with staff drawn from the Department of Health and the Department of Children, Schools and Families. At the regional level, Government Offices and/or Strategic Health Authorities had responsibility for facilitating and coordinating action. Local level action was the responsibility of Primary Care Trusts (PCTs).

*HWHL* ran officially until the end of the spending period in March 2011, but was effectively terminated at the national strategic level in May 2010 following the election of the current Coalition government. Local level actions were generally scheduled to continue up until the reorganisation of local public health delivery in 2013 (which included the abolition of PCTs). The Strategy was formally replaced in November 2011 when a new Strategy, *Healthy Lives, Healthy People: A call to action on obesity in England* was published by the Coalition government [18].

## Methods

### Study design

In-depth, semi-structured, one-to-one interviews were used to explore stakeholder perceptions of the drivers and outcomes of the development and implementation of *HWHL*. This one-to-one approach was adopted to enable interviewees to express their perceptions on what is recognised as a very complex and politically sensitive issue. The aim of the questions was to elucidate perceptions of: 1) the Strategy document, 2) what stimulated the development of the Strategy, 3) outcomes of the development and implementation of the Strategy, 4) elements of development and implementation of the Strategy credited with contributing to positive outcomes, and 5) the challenges and perceived limitations of the Strategy.

### Sampling

A purposeful sampling strategy with maximum variation was adopted to capture a wide breadth of perceptions from individuals involved in the development and implementation of *HWHL* (Table 2). The main aim of the sampling strategy was to identify interviewees who could report first hand on the development and implementation of the strategy. The secondary aim was to identify interviewees who had a stake in the development and implementation of *HWHL* and who could provide an external perception. Six groups of actors were targeted: (1) people responsible for drafting of the Strategy and their advisors, (2) people responsible for implementing the Strategy at the national, regional and local level, (3) leading professional groups and NGOs involved in obesity prevention and management, diet and/or physical activity, (4) voluntary sector organisations with some involvement in Strategy development or implementation, (5) leading actors in the private sector, and (6) the media (Table 2). Priority was given to ensuring adequate representation from the first two groups. 85% of those approached agreed to be interviewed, but there was no response from invited representatives from the retail industry.

**Table 2:** **Participants’ roles in *Healthy Weight Healthy Lives***

**Individuals with lead responsibility for strategy (n = 25)**	**Leading stakeholders in obesity nationally (n = 10)**	**Marginal involvement in strategy (n = 5)**
National Civil Servants (6) Expert Group of Academics (3), Regional Government (4) National Health Service Primary Care Trusts (12)	Non-Governmental Organisations & Professional Organisations (5), Weight Management Community (4), Food Manufacturing Industry (1)	Non-Governmental Organisations (4) Media (1)

### Interviews

Semi-structured one-to-one interviews were conducted by CH between November 2011 and April 2012, either face-to-face (31 interviews) or by telephone (9 interviews). Interviews lasted between 40 minutes and 2.5 hours, typically lasting a little over an hour. Every interview included the same broad categories of open-ended questions on: 1) Strategy development; 2) Strategy implementation; 3) Monitoring and evaluation; 4) Cross cutting issues around key drivers, challenges, impact and lessons learned. Questions from the core set were tailored to each participant taking into account their specific role in the Strategy. The emphasis at the national level was on development followed by implementation, while at the regional and local levels there was a much stronger emphasis on implementation. The focus was on the Strategy as a whole, rather than specific initiatives within the Strategy, although questions were asked to assess whether parts of the Strategy were perceived to be more significant than others, or had been more thoroughly implemented. Questions did not seek to assess how the strategy had impacted on obesity rates.

### Analysis

The interviews were recorded and transcribed. Transcripts were analysed using a thematic framework analysis, which allows the analysis to be guided by specific research questions [19]. After half the interviews had been conducted, the authors began the initial process of reading the transcripts, and met to discuss the most salient observations and to develop the initial coding framework. An initial broad framework focussed on four overarching themes: attitudes towards the Strategy, the perceived outcomes of the development and implementation of the Strategy, the elements of Strategy development and implementation that made these outcomes happen and the limitations of the strategy. Themes and sub-themes within these key areas were identified from the text of the interviews. An iterative process was used to revise the coding matrix as the analysis progressed, with subsequent meetings held to establish consensus. Every segment of text was manually coded into these themes and sub-themes, sub-divided according to differences of perception. The number of interviewees who referred to each theme and sub-theme, was tallied up as a means of identifying the dominant themes and sub-themes, dominant perceptions, and minority perceptions. In a small number of cases, it emerged during the analysis that the interviewee had not responded to particular questions, had answered in a way that was unclear, or had referred to information sources with no details of where it could be found. In these cases, follow-up emails were sent to the interviewees to fill the gaps.

CH led the analysis and AA independently checked and verified emerging themes. All authors then discussed and agreed core themes and sub-themes.

### Ethical approval

Ethical approval for the study was obtained from the University of Cambridge Psychology Research Ethics Committee. All participants gave informed consent. Participant quotes are only identified by role-defined groups and participants had the opportunity to review the manuscript and remove any direct or indirect quotes that could be associated with them. 35 out of the 40 informants responded saying they were happy with the manuscript, with a small number providing some specific additional comments. None requested any quotes be changed or removed.

### Roles of the authors

CH was the only author to conduct the interviews. She was personally known to 6 of the interviewees, but she was not involved in the development or implementation of *HWHL*. AA is a research scientist in the obesity field and also had no involvement in the strategy but was personally known to 3 of the interviewees. SAJ is a government advisor on obesity and was herself interviewed as part of this analysis. She was personally known to 23 of the other interviewees, of which most were those involved in the development or implementation of the Strategy at a national level. She did not participate in the identification of themes from the interviews but contributed to the discussions about the overall findings and drafting of the paper, in particular the wider context for the Strategy. Participants were informed that what they said was confidential and would not be used to identify them. The range, depth and detail of participant responses suggest that they did not feel constrained by their knowledge of the research team. The knowledge and experience of the research team enabled the interviews to be contextualised and facilitated understanding between the interviewees and the researchers. During data analysis the team adopted strategies to reflect on how their interpretation of the data could be influenced by their own experience and challenged each other when they felt that assumptions were being made which were not directly evident in the transcripts.

## Results

### Overview

Informants had clear perceptions of what drove the development of the Strategy, of the final document, and the perceived outcomes and elements responsible. Interviewees identified four key differences that *HWHL* made to efforts to address obesity in England: political buy-in and multi-stakeholder engagement to a national obesity strategy, more action taken to address obesity at all levels, other government departments more engaged with obesity as a serious public health problem and positive changes in awareness and attitudes about the role of government in addressing obesity (Table 3). Five key elements of *HWHL* emerged from the interviews as being critical in leading to these positive outcomes (Table 3). These were the multi-faceted, inclusive nature of the strategy, the governance structures set up to implement cross-government working, the obesity target and systems to measure progress against the target, the focus on children and ring-fenced funding.

**Table 3:** **Lead themes identified on perceived outcomes of *HWHL *and the elements perceived as responsible**

Perceived positive outcomes	• Political buy-in and multi-stakeholder engagement with a national obesity strategy
	• More action taken to address obesity at all levels
	• Other government departments more engaged with obesity as a serious public health problem
	• Positive changes in awareness and attitudes about the role of government in addressing obesity
Perceived limitations	• Actions in other government departments were relatively limited
	• Inadequate learning about the effectiveness of the different elements and actions in the Strategy
	• Insufficiently tough on the food industry
	• Insufficient attention paid to treating the significant population who are obese, especially adults
Key elements responsible	• Multi-faceted, inclusive nature of the strategy
	• Governance structures set up to implement cross-government working
	• Obesity target and the means of measuring progress against the target (National Child Measurement Programme)
	• Child-focus
	• Funding

### Strategy development

The decision to develop a cross government obesity strategy was in part attributed to a favourable political climate. The British Prime Minister had expressed concern about obesity, it was a priority of the health minister at the time, who was considered a key player in the governing party, the children’s minister considered schools an appropriate place to deliver public health objectives, the government as a whole was focused on meeting social policy goals and money had already been allocated to obesity in the spending review.

“I think the thing that facilitated [the development of a Strategy] was having a ……..sort of strong sense of common purpose, you know, … (the) minister’s sense of a need to really sort of get on top of [obesity] as an issue.” *(national civil servant)*

Interviewees felt the Strategy was timely– a range of actions to address obesity had already been taken, but there was no Strategy in place to develop a joined-up approach.

The most widely cited stimulus to the HWHL Strategy was the Foresight report on obesity. This comprehensive overview of the scientific evidence was credited with providing the wake-up call that greatly increased the imperative to act.

“I would say that [the Foresight obesity report] was the real lightning rod because I could just remember [the health minister] saying, “We have to respond to this report. What are we going to say because what we’re doing at the moment isn’t enough?” *(national civil servant)*

The civil servants responsible for developing the strategy said they looked directly to the evidence compiled by Foresight for guidance. They also sought the advice of an Expert Advisory Group of academics who advised on Foresight, and who were later established as advisors to *HWHL*. Those responsible for drafting the Strategy said the Foresight report encouraged them to draft a Strategy that: (1) provided a framework to bring everything together (2) addressed the environmental determinants of diet and activity (3) incorporated a wide range of stakeholders and (4) was informed by existing evidence as much as possible.

### Overall perception of the strategy document

Thirty-nine of the 40 interviewees said that having a national strategy added value to previous efforts to address obesity, and 36 described the Strategy in predominantly positive terms. Those responsible for implementing the Strategy praised the way *HWHL* provided a big picture, inclusive framework that brought all the different elements of the problem together.

“It was useful to have a document that laid out almost in some ways some kind of structure of everything you’d need to tackle.” *(PCT, obesity lead)*

Interviewees said they felt that it identified the roles and responsibilities of the different stakeholders – making people realise they all had to work on it together.

“You know, it’s a complex and multifaceted issue that needs to be tackled in that way and I think that that strategy helps everybody who is involved think in that way and thinking about their contributions.” *(PCT, obesity lead)*

Local and regional interviewees also described how it gave a detailed steer on the actions needed and how to prioritise those actions.

“I think it was really useful, and it did give a very detailed list of how to go about things. I mean you couldn’t really go wrong with the strategy. It had thought, had done a lot of the thinking for you, and had you know, some good practice examples. ” *(PCT, obesity lead)*

The Strategy also received support from NGOs, largely on the basis that it included the “environmental determinants” of obesity.

“It wasn’t just about lifestyles, it was a comprehensive programme that dealt with the wider social determinants”. *(NGO, director)*

The food industry informant said the Strategy was supported by the private sector because it acknowledged obesity as a complex problem requiring action by a wide range of stakeholders, including individuals.

While the perception of the Strategy document was very positive overall, some interviewees voiced criticisms of the content. Three interviewees felt that the Strategy had not gone far enough in translating the comprehensive, systems approach taken by the Foresight report. Thirteen local informants and NGOs said the Strategy was insufficiently tough on the food industry. Four of the interviewees involved in weight management felt that the very dominant prevention-focus of the Strategy was a major problem.

“I thought the emphasis was too much on prevention….I absolutely understand that … you want to treat the cause not the symptom. But at the same time when the symptom has a very human face to it and has a, very significant numbers attached to it, it seems both short-sighted and pretty irresponsible to only look at treating the cause and not the symptom, you need a good balance of both.” *(weight management programme, director)*

Two of these informants said that they provided input and evidence during the development phase of *HWHL*, but felt their views were not adequately represented in the Strategy. Weight management programmes were also unhappy with the poor execution of the plan to develop a national framework for commissioning weight management programmes. This was also acknowledged by interviewees with experience of the process at the Department of Health. The focus on prevention was also blamed for a lack of engagement with the Strategy by health professionals, especially clinicians.

### Impact of multi-faceted, cross-government approach taken by *HWHL*

The multi-faceted, inclusive approach of the Strategy was the dominant element perceived to have led to positive outcomes on efforts to address obesity in England. It was widely praised and credited with creating political buy-in and facilitating cross-government engagement with the Strategy. Civil servants reported that the process of developing a cross-government Strategy provided them – and the health minister – with a platform from which to discuss roles and responsibilities with colleagues in other parts of government. It was said to have facilitated sign-off by the relevant cross-government Cabinet committee (a process required for all government strategies in England), thereby enabling *HWHL* to become official government policy. National officials described the process of engaging other departments during the development of the Strategy as challenging, but said they were more-or-less satisfied with the outcome.

“I think what *Healthy Weight, Healthy Lives* did was bring more people to the fold as it were and shift the focus a little bit away from [the department of] health. It didn’t achieve total cross-government ownership but I don’t know any issue that does to be honest.” *(national civil servant)*

The strongest message about cross-government engagement during implementation came from the regional and local level. These interviewees said that *HWHL* gave them the push, impetus and legitimacy to start, or significantly expand, their cross-government engagement.

“[The Strategy] did give us some levers to start talking to different directorates or departments within local government …Off the back of this, we started working more closely with planning and with transport.” *(PCT, obesity lead)*

Two interviewees went further, stating that the cross-government engagement initiated as a result of *HWHL* provided the groundwork for more collaborative working on obesity. This issue was noted as particularly significant in the light of the shift in the public health functions of PCTs into local government in 2013, as a result of Coalition Government policy.

### Impact of cross-government governance structures

The Strategy was implemented through a series of cross-government governance structures, including those at Ministerial level. These were credited with facilitating engagement at all levels, including the creation of cross-government teams for steering and/or implementation of policies, and an opportunity to engage with the various stakeholders.

These include:

• Ministerial Committee on Domestic Affairs, Sub-Committee on Families, Children and Young People, and Sub-Committee on Health and Wellbeing (both comprised of ministers from a range of departments)

• Cross Government Senior Officials Group

• Cross Government Obesity Unit between the Department of Health and the Department of Children, Schools and Families, reporting to a Programme Management Board

• Cross-government teams and/or steering groups in Regional Offices of the Department of Health and/or Strategic Health Authorities (sometime also a sub-regional structure)

• Joint staffing appointments between government departments

• Joint targets at the national, regional and local level

These structures were also credited with driving action in sectors outside of health. This was particularly the case for actions taken by education and children’s departments, which were responsible for the actions reported most frequently by the informants as part of the Strategy. At the national level, the emphasis on actions in schools and for children was attributed to the shared responsibility between departments.

“The stuff around schools and Healthy Schools, we wouldn’t have been able to do… anything that kind of isn’t within our direct responsibility.” *(national civil servant)*

A similar picture emerged at the regional and local levels:

“[Cross-government working] sort of harnessed our ability to pull in all these various inputs that we needed to mobilise, to actually get them focused on obesity as a priority and see where their contribution was so, you know, school travel advisor who maybe hadn’t worked so collegiately with the Department of Health on public health priorities was a key member of how we delivered a number of initiatives and interventions around obesity, same with Healthy Schools.” (Regional obesity lead)

Three informants – from a regional government office, a sub-regional position and a PCT – said that they had developed joint targets with different departments (independently of national government). These were said to have stimulated action in other departments by creating shared agendas.

“[We received] a very positive reception [from the children’s department]. Of course the Healthy School targets were ones they wanted to achieve [and] because those targets were the stretch targets so they had a financial reward, the possibility of a financial reward.” *(PCT, obesity lead)*

National government, regional and local entities also reported success stories in cross-government working on issues of active travel and green spaces, which was attributed to shared policy objectives:

“It’s that classic thing of trying to express your priorities in terms of their own priorities so that they at least buy in to what you’re doing and, say for DfT [Department of Transport], that was really easy because we all wanted to shift people from cars to kind of bikes and walking. It’s exactly what they want to do, exactly what we want to do, it was fine.” *(national civil servant)*

However, it was widely reported that cross government actions were generally limited to specific areas of work, and it was often hard to get beyond dialogue. Nationally and locally, informants said the onus of responsibility remained with the health department:

“It does still feel like it’s health going and asking about, “Please consider the impact on health when you make this decision”, rather than other departments, like I say, planning or leisure or whoever, not coming or, you know, the food industry or whatever, coming and saying ‘help us, we want to make sure that we don’t negatively affect the health of the population’. So that hasn’t changed as a result of this so … maybe it sowed a seed but…” *(PCT, obesity lead)*

Actions were said to become more limited as soon as interests diverged. This was widely reported in the case of planning. At the local level, all except one of the PCTs reported they had approached their planning departments, but the actions that were taken as a result were limited (the remaining PCT did not approach their planning department because it “would have been too big a mountain to climb”). The reasons cited were different interests and inadequate incentives for the planning department to get involved. This was even the case where there was a planning representative on the regional obesity team and a joint staffing appointment.

“It started to get difficult because they obviously had their own things that they do, their own priorities and their own objectives, and child obesity doesn’t really feature in those.” *(national civil servant)*

In fact, outside government, the extent to which the Strategy really led to cross-government action was widely questioned – a common perception was that the Strategy was really a Department of Health initiative, with some work with the Department for Children, Schools and Families and little else. It was notable that the media informant said they perceived *HWHL* as a Department of Health rather than cross-government initiative because it was always the Health Minister who made announcements and did the media interviews.

### Impact of monitoring against national and local targets

*HWHL* was developed in the context of a national government target for 2020. Interviewees said they recognised the need for a long-term target owing to the long-term nature of action to address obesity. However, 16 informants commented on the inadequacy of having a single long-term target, and 12 specifically said they thought additional shorter-term targets, indicators or process-indicators “with teeth” would have helped drive further action within the timetable of political cycles (though they also recognised the challenges in measuring these types of indicators). Two interviewees said there should have been a target for adults as well as children.

In some local areas the national target was used to develop specific and shorter-term obesity targets. Setting these targets was not mandatory, but many local authorities chose to develop one [20]. These targets were tracked using the “child obesity indicators” from the Local Government National Indicator Set [21].

A significant number of interviewees [19] said the national and local targets had made a positive contribution towards the implementation of the Strategy. At the national level, the presence of targets was said to demonstrate to others that the government was serious about tackling obesity and attracted the funding from the Treasury. Locally, informants said the targets focused the minds of politicians and senior officials on obesity. Most critically, at all levels, they were said to create an incentive to act:

“[The target was] vital, yes. Because without a target no one would have done anything.” *(Expert Advisory Group, member)*

Even more than the target, informants emphasized the importance of measuring progress against the target as the key element that stimulated action:

“If we’re performance-managed against it, it does tend to focus people’s minds, and money.” *(PCT, obesity lead)*

The reporting process was the National Child Measurement Programme (NCMP), set up prior to the publication of *HWHL*. Informants said the data produced by the NCMP was crucial in providing the evidence to local level officials, especially at a senior level, that obesity was actually a problem.

“I think the performance management was around getting senior leadership focused, I think that’s the, that’s where [the NCMP] helps, definitely.” *(sub-regional obesity lead*)

The data was also said to enable local areas to identify the areas of greatest need.

“We’re now proactively following up children via the school nurses’ service and using the NCMP for data and to build up a picture of childhood obesity prevalence” *(PCT, obesity lead)*

By providing the evidence on whether the prevalence of obesity was increasing or decreasing in every local area across England, regional and local level informants made it clear they knew they were being judged against the target:

“We wait with bated breath every year for the figures to come out.” *(Director of Public Health)*

Informants also highlighted the role played by the independent entity the National Obesity Observatory (NOO), which was tasked with supporting local areas in obtaining and understanding the NCMP data.

“[NOO] has been an enormous benefit to everybody, you know, the fact that you can just go to that one site and look up the evidence and data is just great.” *(PCT, sub-regional obesity lead)*

Informants said that the NOO was the first place they went to obtain the data they needed from the NCMP to support their work. Regional and local level informants said NOO’s synthesis of the data filled a major gap, since they would never have had time to do it themselves.

### Impact of child-focus

Interviewees frequently discussed the Strategy’s focus on safe-guarding the lives of children. Most of these interviewees (16 out of 23) were favourable to the child-focus. This was because they saw it as justified in public health terms and because they perceived it as critical in getting political buy-in.

“There was more legitimacy to be seen to protect the health of children, rather than interfering if you like…. government interfering with the life choices of adults.” *(national civil servant)*

The focus on children was also said to facilitate relationship building by giving an ‘in’ to other departments:

“The focus on children was good from the perspective of actually building relationships and collaboration with the Department for Children, Schools and Families, which was critical in terms of the success of the programme because, again, it got us in dialogues with Directors of Children’s Services, as well as Director of Public Health; got you into the local authority realm; got you into their performance management regime systems to their priorities, conversations, you know.” *(national civil servant)*

Nine informants – seven from regional and local areas – said that the local targets were a leading reason why so much of the action focused on children than adults.

“Yeah. Well I think the children, the focus on children came about because the target that was set for, you know, the outcome in terms of reducing child obesity.” *(regional obesity lead)*

Those least likely to favour the child focus were weight management professionals:

“I think a policy needs to have prevention and treatment across all the ages, but you obviously need to target those at of highest risk. But I think those adults who are already obese, or already overweight, were very much ignored within this strategy.” *(weight management stakeholder)*

The child focus of the Strategy was the reason cited by civil servants that the “Creating incentives for better health” stream of *HWHL* had not been adequately implemented (Table 1). This stream was predominantly concerned with engagement of employers and organisations, and thus targeted adults. According to those involved with implementing the strategy at the national level, this adult focus made it feel less central to the overall strategy.

### Impact of funding

Twenty-six of the interviewees spoke at some length about funding. Sixteen of these said funding was a critical element in implementing action, seven of whom were referring to national funding, and nine of whom were referring to regional and/or local funding.

“*HWHL* had a lot of money attached to it and that makes a difference. Before, [officials in government with responsibilities for obesity] hadn’t had very much.” *(national civil servant)*

Funding was credited with enabling three particular processes: a comprehensive structure and dedicated staff to implement the strategy, incentives for cross government action (when funding was provided to other departments), and specific new initiatives. For example funding supported a large social marketing campaign, the National Obesity Observatory and a range of programmes regionally and locally.

“It, well we got considerable extra funding as a result of it into our budgets and that made a big, big difference and enabled us to… to do a lot of things that we had never been able to do before. And secondly, it enabled us to put in place a process to encourage public health in the local area to operate effectively…” *(Director of Public Health, Regional Government Office)*

However, one sub-regional and one local level informant said that funding was actually hard to get – that they had to fight for what was available, suggesting a range of different experiences in practice.

Five informants noted that they thought funding was not the most important thing – that much could still have been done with less funding.

“You probably don’t even need that much money, you could do it with less money than we had, although money always helps and it probably means that you can get things going quicker.” *(national civil servant)*

### Impact of learning mechanisms

At the national level, civil servants said learning and evaluation were taken very seriously and a wide range of different mechanisms was set up to do so.

These included:

• Childhood Obesity National Support Team – provided tailored support on invitation to local areas

• Regional meetings of local obesity leads – shared experiences and learnings from local level action

• National annual progress reports – provided overview of actions taken [20]

• Evaluation of specific national initiatives – such as Healthy Towns and Change4Life [10]

• Evaluations of specific actions at the local level

• Development of standard evaluation frameworks – to provide guidance for evaluation of weight management interventions by NOO* [22]

• Funding of the Obesity Learning Centre – an Internet-based resource [23].

*Since the termination of *HWHL*, standard evaluation frameworks have also been developed for diet and physical activity interventions [22].

Of the learning processes, two were said to be particularly useful at the local level: the regional level meetings of local obesity leads and the support provided by the National Support Team (now dissolved). Both structures were said to help build knowledge and improve local strategies and practices.

“[Regional networks] just facilitated everybody at a regional level getting together and sharing good practice and I think that was really, really useful.” *(PCT, obesity lead)*

The nine informants with experience of the National Support team said they thought local areas developed better local strategies as a result:

“Well, I mean, I think it’s another pair of eyes, a critical friend, a look at areas where they needed to improve upon in terms of delivering on *Healthy Weight, Healthy Lives* and I think everyone that participated in that found it incredibly useful.” *(Regional obesity lead)*

In terms of evaluation, the Department of Health funded evaluations of some of the key initiatives [10], and ten of the regional/local area informants said they evaluated some of their own initiatives. Five said they found the Standard Evaluation Framework for weight management programmes useful.

Nevertheless, 20 informants reported that evaluation was inadequate and difficult. They said that as a result of this, *HWHL* had not produced adequate learning about the effectiveness of different elements and actions in the Strategy. Interviewees offered various different theories about why the evaluation process had not led to the intended outcomes. These included the absence of a culture of evaluation at the national level, problems with evaluations of specific programmes [10], failure to build in evaluation at the programme design stage, the misperception that the NCMP was sufficient to evaluate the effects of specific initiatives, and genuine scientific uncertainty and complexity around the appropriate intermediate indicators/metrics to measure the effect of obesity initiatives. Three interviewees said there had been inadequate funding for evaluation, and six noted lack of time. The Strategy was implemented over a relatively short time period, and there was also said to be too little time to set up evaluation processes before the interventions began.

“What we needed was more time, time to allow the evaluations, time to allow the evidence base to build.” *(NGO, Director)*

The most clearly articulated reason for the problems with evaluation (a reason offered by 11 of the interviewees) was the lack of a national framework of performance indicators or metrics to guide evaluation and the lack of a systematic mechanism for feeding back and collating results at the national level. This was said to reduce the consistency and comparability in evaluation and reporting across areas.

“It’s something where there’s a real role for a national framework and support, because the more you’ve got common indicators, the more you can do comparative work with other areas… and glean broader learnings.” *(PCT, director)*

As a result of these missing elements, interviewees expressed frustration that they were not able to judge if the different policies and programmes in *HWHL* had made a tangible difference to obesity prevalence in England.

“If we can’t demonstrate success because we’ve not come up with criteria for judging it and measured against ourselves, other than [long-term obesity] targets, then, you know, I think we’ve not learned a great deal.” *(professional association, official)*

### Overarching outcome

Informants were specifically asked about what they perceived the outcomes of *HWHL* to be as a whole. The most commonly cited outcome (by half the interviewees) was that it raised the profile and significance of obesity among decision-makers.

“The nature of the problem hasn’t changed. The problem has been severe for years, but the profile and the commitment and the visibility to do something about it I think is higher now than it has been and was before.” *(national civil servant)*

Interviewees said *HWHL* changed attitudes towards obesity within government by showing that government does have a role to play and that cross government work is essential.

“[The Strategy] pulled together for the first time in a really clear way that government does have a role in trying to help people make choices in this way. And that’s still, now, I think, whatever the political climate, that is still the approach that is recognised.” *(NGO, senior staff member)*

## Discussion

### Strengths and limitations of the research

The current study sought to qualitatively explore the experience of developing and implementing a national obesity strategy. It provides insights into the less tangible but potentially critical impacts of the strategy that cannot be captured by quantitative measurements. The personal perspectives of stakeholders directly involved in the development provided unique contextual information and insight into the thinking that informed the Strategy. The study design and interpretation of the data sought to elicit a diversity of opinions and experiences by including both the lead actors involved in the development and implementation of the Strategy, and a range of other stakeholders with varying levels of involvement in the strategy and different roles, perspectives and priorities.

This research used retrospective interviews, which asked participants to recall their experience of the strategy over a period of several years. This analysis therefore reflects how participants have retrospectively contextualised and interpreted their experiences. Their personal involvement in the strategy may have influenced their comments about *HWHL*, and this should be considered in any interpretation of these findings. In addition, some stakeholder groups were not represented, or only in small numbers. Likewise, it is important to consider that the knowledge and experience of the authors could be perceived both as a strength, in that it facilitated more in depth interviewing and analysis, and as a limitation, in that it may have influenced the selection of participants, choice of questions and the focus of the analysis.

### Insights for the development and implementation of population-based obesity strategies

*HWHL* satisfied many of the conditions required for a population-based obesity strategy. It took a multifaceted approach*,* addressed the breadth of causes of obesity, involved a range of government departments and sectors during both development and implementation and it was implemented at multiple levels of government [5]. According to the informants in this study, it was also a politically popular Strategy which stimulated cross-government engagement; lay the groundwork for cross-government action, and enhanced knowledge and attitudes about obesity and obesity prevention within government. This yields the insight that developing a Strategy which incorporates roles for all stakeholders and the multiple determinants of obesity at a population-level can create political buy-in. Basing the Strategy on the best-available evidence – which indicated a multi-faceted strategy was necessary – and the focus on children also appeared to help overcome concerns about “nanny statism” and scepticism that it is possible for the government to positively to change people’s behaviours [24]. The findings from these interviews suggest that inclusiveness, the weight of the evidence and the political and public will to safeguard children acted to counterbalance political anxieties about excessive government control over individuals food and physical activity choices.

A second insight is that establishing governance structures for cross-government engagement is necessary but not sufficient to stimulate adequate cross-sectoral action [6]. Cooperation is relatively easy where there are already shared agendas (e.g. joint policy priorities, shared targets and funding), but not where there are contrasting or competing interests. This finding has also been found elsewhere [25-29]. International experiences of successes and failures in multisectoral action in nutrition and public health suggest that time spent investing in evidence-gathering to identify shared goals, objectives and agendas may well have produced better outcomes [28]. It also indicates that success requires the stakeholders to identify a *need* to work together to achieve their goals – and for there to be clear incentives to do so [27,29,30].

In *HWHL*, there was no evidence that a systematic process had been followed to identify shared needs, goals, objectives and agendas, or to develop incentives to create them. Nor were specific plans to monitor and evaluate the *cross-government* aspect of the Strategy put into place. Accordingly, *HWHL* could not be described as adhering to the principle to “plan multisectorally, implement sectorally, review multisectorally*” *[29]. This implies that a more rigorous process is needed for successful cross-government engagement, including clearer mechanisms for collaboration, such as joint targets, reporting structures, and financial incentives. These findings echo that of a recent study of efforts to tackle health inequalities in the UK, which found that cross-government working was more aspiration than reality, and requires powerful forces to make it happen [31].

A third insight from these interviews is the perception that setting targets and having a means of monitoring them provided a strong incentive for government stakeholders to implement actions to address obesity. While health targets have been shown to be problematic when they place unrealistic demands on implementers [32,33], interviewees in this study thought that targets were helpful in stimulating action. The target and the monitoring programme appeared to provide England with a system of public accountability for childhood obesity (which the current government has opted to continue, despite considerable changes in public health since the Strategy). This finding supports the WHO recommendation that governments adopt an obesity target [34], even if it is aspirational. However, it also suggests that such a target will only lead to action if it is accompanied by a system of monitoring and regular reporting.

A fourth insight concerns the learning processes in *HWHL* about the effectiveness of different elements and actions. It is evident that evaluating population-based obesity strategies like *HWHL* is very challenging owing to the wide range of interacting actors, activities and settings [35]. Monitoring against a target is not sufficient to identify what actions within the strategy are effective [36]. Rather, greater efforts are needed to place evaluation at the heart of population-based obesity strategies. Strategy developers and implementers should focus harder on generating new evidence for the future by building in and operationalising a framework for learning and evaluation from the very beginning.

## Conclusion

This study assessed the perceptions of stakeholders about the value of the *HWHL* Strategy in efforts to address obesity in England and to identify insights that could be used to inform the development and implementation of future obesity strategies in England and elsewhere. It found that the Strategy facilitated political engagement with the obesity problem, stimulated action, and changed knowledge and attitudes. The key elements responsible for these positive outcomes were the multi-faceted, inclusive nature of the strategy and accompanying governance structures, a focus on children, funding and the presence of a target and a mechanism to monitor progress against the target. The development of the Strategy itself was stimulated and aided by the effective synthesis of critical mass of scientific evidence.

The English experience of *HWHL* lends support to recommendations to develop population-based obesity strategies. It indicates that in order to stimulate comprehensive action, and for that action to take place across sectors, obesity strategies need to (i) take a population-based, multi-faceted approach implemented through a clear governance structure, (ii) involve a systematic process of aligning goals, objectives and agendas between government departments and sectors with stake in obesity, and (iii) have a clear system of reporting changes in obesity rates against a target. In order to design effective policies to tackle obesity and to build the case for continued investment, obesity strategies also need to incorporate a national framework for learning and evaluation from the outset.

## Competing interests

CH received funding from MRC Human Nutrition Research to conduct the study; CH was a member of the National Obesity Observatory eTool project advisory group; SAJ was the Science Advisor to the Department of Health on Obesity from 2007–2013, and between 2007–2011 she was Chair of the Department of Health’s Expert Advisory Group on Obesity. She is currently Chair of the Public Health Responsibility Deal Food Network.

## Authors’ contributions

CH developed the methods, conducted all the interviews, coded the interviews, identified themes and drafted the paper. AA contributed to developing the methods and study design, including the questionnaire, read the interview transcripts, and independently checked and verified the emerging themes identified by CH. AA also made a significant contribution to the structure and content of the paper through reviewing drafts. SAJ conceived the idea for the study, contributed to study design with regard to the interviewees and the questionnaire, made the primary contact with several of the informants, provided input into the interpretation of the emergent themes, and made a significant contribution to the structure and content of the paper through reviewing drafts. As a member of the Expert Advisory Group to the UK Department of Health at the time of Healthy Weight, Healthy Lives, SAJ was also interviewed as part of the study. All authors met to discuss findings, but CH and AA were responsible for the final judgments on interpreting the interviews. All authors approved the final version of the paper.

## Pre-publication history

The pre-publication history for this paper can be accessed here:

http://www.biomedcentral.com/1471-2458/14/441/prepub
